# A Rare Case of Spermatic Cord Metastasis Following Surgery for Transverse Colon Cancer

**DOI:** 10.7759/cureus.80679

**Published:** 2025-03-16

**Authors:** Ryo Sato, Shungo Kakinuma, Shunsuke Mochizuki, Asuka Uchiyama, Shiori Meguro, Rikiya Matsumoto

**Affiliations:** 1 Department of Urology, Chutoen General Medical Center, Kakegawa, JPN; 2 Department of Urology, Hamamatsu University School of Medicine, Hamamatsu, JPN; 3 Department of Pathology, Chutoen General Medical Center, Kakegawa, JPN; 4 Department of Regenerative and Infectious Pathology, Hamamatsu University School of Medicine, Hamamatsu, JPN

**Keywords:** metastasis, oncology, spermatic cord tumors, transverse colon cancer, urology

## Abstract

We report a rare case of spermatic cord (SC) metastasis in a 44-year-old man with a history of transverse colon cancer. He had undergone laparoscopic right hemicolectomy followed by adjuvant chemotherapy and remained under routine follow-ups with no signs of recurrence. He presented with painless right scrotal swelling, and imaging revealed an SC tumor (SCT). Serum tumor markers were within the normal ranges. Radical orchiectomy was performed, and histopathology confirmed metastatic adenocarcinoma from the transverse colon. Local recurrence was detected four months postoperatively, and the patient is currently receiving multidisciplinary treatment. Metastatic SCTs, although rare, need to be considered in patients with a history of gastrointestinal cancer, even without elevated tumor markers.

## Introduction

Metastatic tumors of the spermatic cord (SC) are extremely rare. The most common primary sites include the gastrointestinal tract, particularly the stomach and colon, followed by the liver and kidneys [1]. Metastases originating from the prostate and pancreas have also been reported [2,3]. Due to their rarity, SC metastases pose significant diagnostic and therapeutic challenges.

The clinical presentation of metastatic SC tumors (SCTs) is often non-specific, with symptoms such as painless scrotal swelling or an inguinal mass, often mimicking benign conditions such as hydroceles or hernias [1]. Given the lack of distinct imaging features and the frequent absence of elevated tumor markers, preoperative differentiation between primary and metastatic SCTs can be difficult. In many cases, a definitive diagnosis is only established postoperatively through histopathological and immunohistochemical analysis.

Here, we present a case of SC metastasis in a 44-year-old man with a history of transverse colon cancer, who initially presented with painless right scrotal swelling. The diagnostic challenge in this case was heightened by the absence of recurrence on routine surveillance imaging, normal serum tumor markers, and the rarity of SC metastases from colorectal cancer.

## Case presentation

A 44-year-old man was referred to our department from a local urology clinic due to a one-month history of painless right scrotal swelling. Three years prior, he had undergone laparoscopic right hemicolectomy for transverse colon cancer. The pathological stage was classified as pT4aN3. He received a six-month regimen of oxaliplatin and capecitabine as adjuvant chemotherapy, followed by routine surveillance. During the follow-up period, there was no evidence of recurrence.

**Figure 1:**
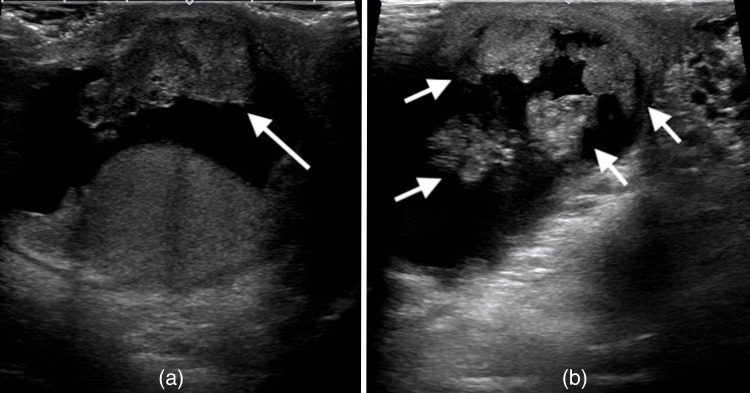
Ultrasonography of the abdomen. (a) A 33 mm mass (long arrow) in the right inguinal region. (b) Several nodules (short arrows) in the right inguinal region.

**Figure 2:**
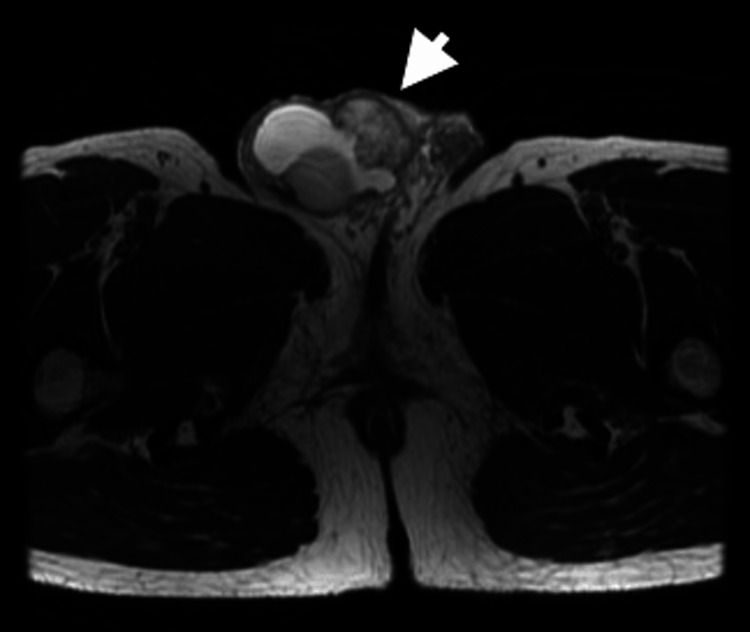
Magnetic resonance imaging (T2-weighted images) of the abdomen. Magnetic resonance imaging shows a tumor (arrow) with a low-intensity signal in the right spermatic cord.

On a physical examination, the right scrotum was swollen to the size of a hen’s egg. A firm, thumb-sized mass was palpated in the right inguinal region. Laboratory tests showed the following tumor marker levels (with their respective normal reference ranges): lactate dehydrogenase (173 U/L; normal range: 124-222 U/L), human chorionic gonadotropin (<1.0 mIU/mL; normal range: <2.7 mIU/mL), alpha-fetoprotein (2.3 ng/mL; normal range: <10.0 ng/mL), carcinoembryonic antigen (1.22 ng/mL; normal range: <5.0 ng/mL), and carbohydrate antigen 19-9 (17.86 U/mL; normal range: <37 U/mL). Ultrasonography revealed a hydrocele, but showed a well-defined, homogeneous right testis with no vascular abnormalities. A 33 mm heterogeneous solid tumor was noted in the right inguinal region, along with several smaller nodules (Figure 1). A 40 × 31 mm mildly enhancing mass in the right SC was observed on computed tomography (CT), with no evidence of recurrence or metastasis in the gastrointestinal tract, liver, lungs, or lymph nodes. Magnetic resonance imaging revealed a low-intensity signal in part of the SC on T2-weighted images (Figure 2). Based on these findings, the preoperative diagnosis was a right SCT with a right hydrocele. The patient underwent right radical orchiectomy under general anesthesia.

**Figure 3:**
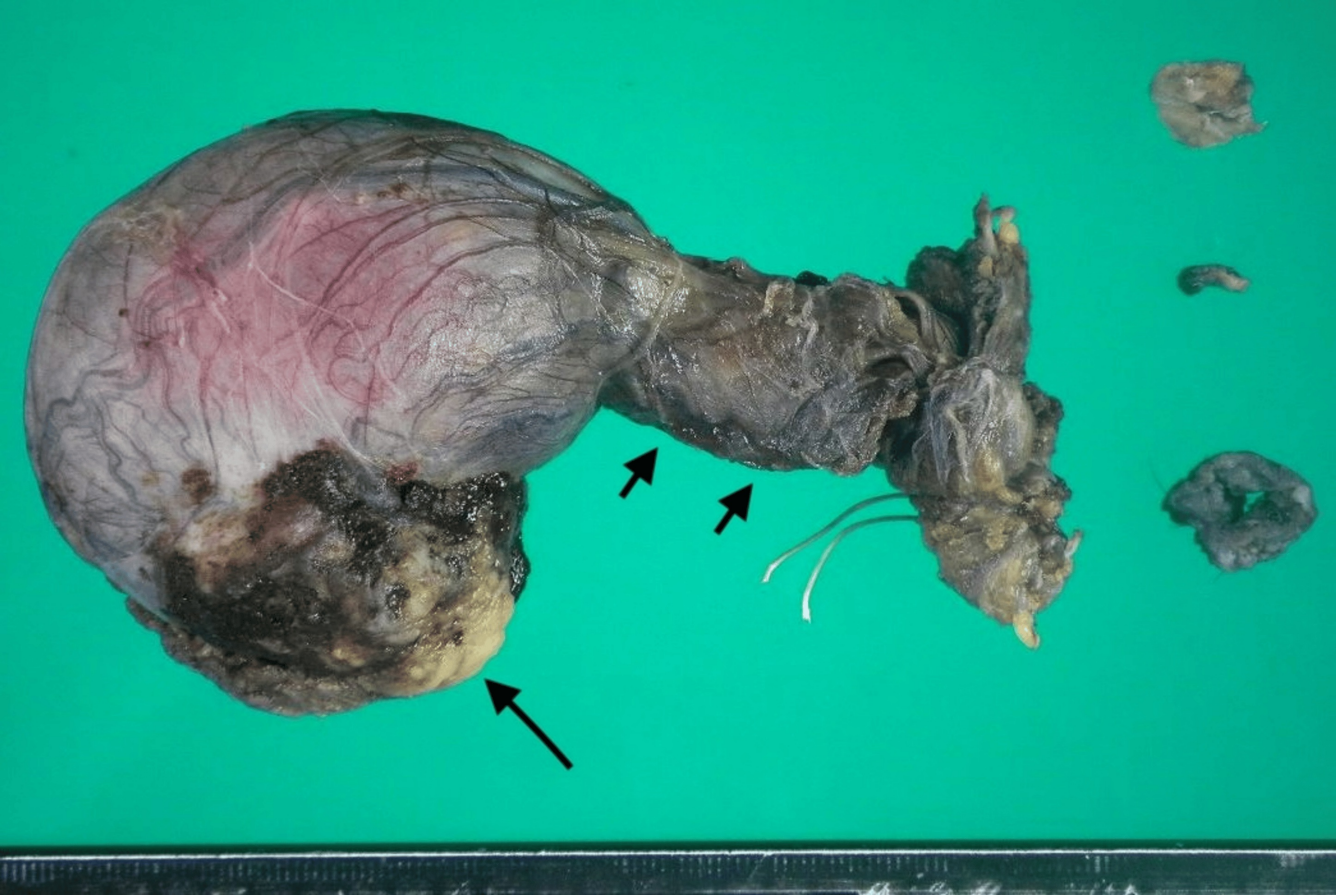
Gross appearance of the resected tumor. Gross examination shows a yellowish-white mass (long arrow) along with multiple smaller yellow-white nodules (short arrows) on the cut surface of the resected tumor.

The tumor strongly adhered to the surrounding tissue; however, it was sufficiently dissected up to the internal inguinal ring, and the right SC was ligated and transected. The scrotal contents were everted, and the entire mass was excised en bloc. The resected specimen contained an ill-defined, yellowish-white, solid tumor measuring 35 × 28 mm within the SC, along with multiple smaller yellow-white nodules (Figure 3).

**Figure 4:**
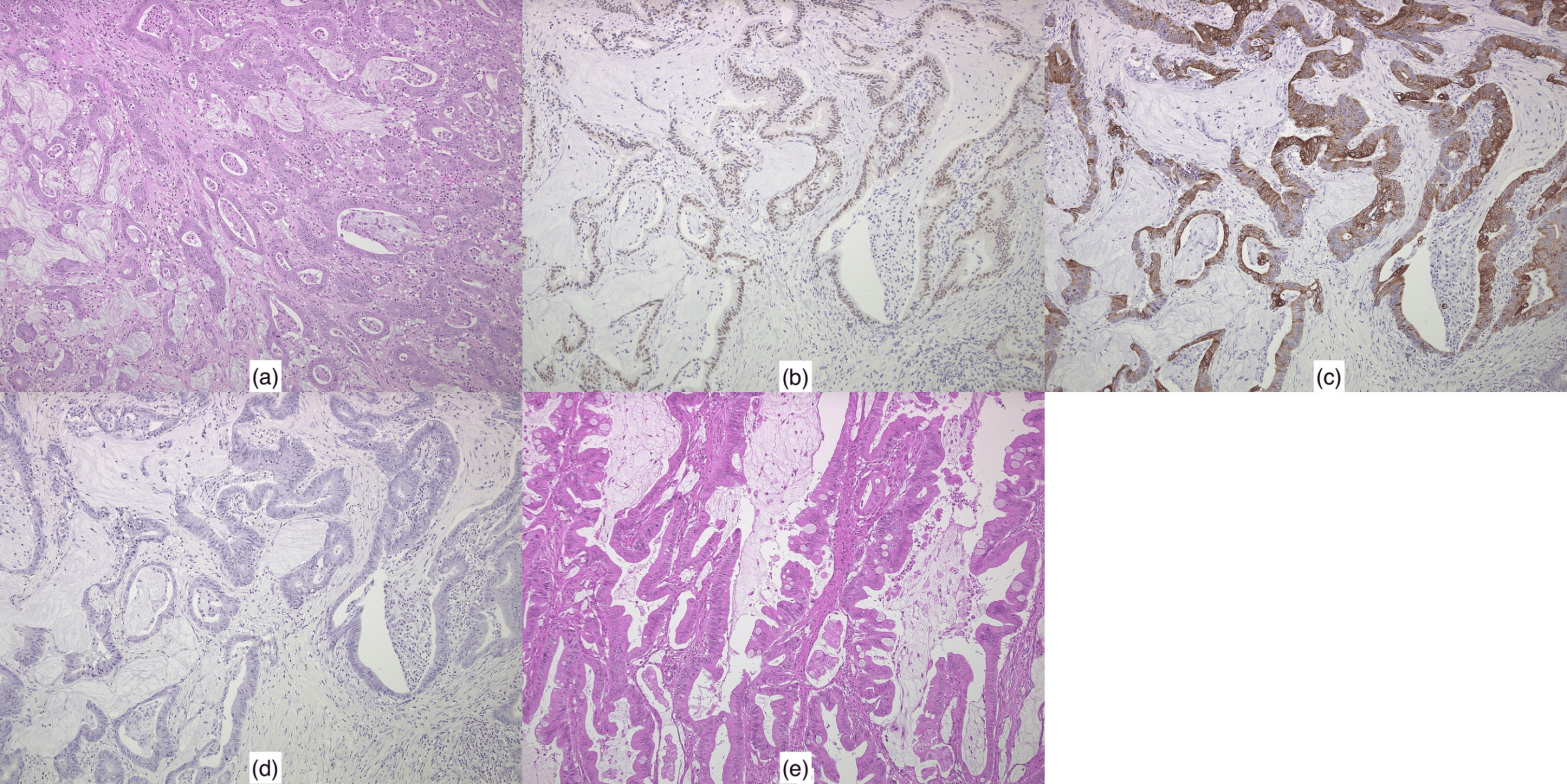
Histopathology findings. (a) The spermatic cord tumor shows moderately differentiated adenocarcinoma, consistent with metastasis from the transverse colon cancer. (b) The spermatic cord tumor shows positive staining for caudal-type homeobox 2 on immunohistochemical analysis. (c) The spermatic cord tumor shows positive staining for cytokeratin (CK) 20 on immunohistochemical analysis. (d) The spermatic cord tumor shows negative staining for CK7 on immunohistochemical analysis. (e) The primary transverse colon cancer exhibits moderately differentiated adenocarcinoma.

A histopathological examination revealed moderately differentiated adenocarcinoma with infiltrative growth in a fused glandular or cribriform pattern, with cells exhibiting enlarged pleomorphic nuclei proliferating with necrosis (Figure 4a). On an immunohistochemical examination, the tumor cells in the SC were stained for caudal-type homeobox (CDX-) 2 (Figure 4b) and cytokeratin (CK) 20 (Figure 4c), but not for CK-7 (Figure 4d). The SC surgical margin was positive. A comparative analysis with the patient’s previous transverse colon cancer specimen (Figure 4e) revealed morphological similarities, which, in conjunction with the immunostaining results, ultimately confirmed the diagnosis of SC metastasis from transverse colon cancer.

The postoperative course was uneventful, and the patient was discharged on postoperative day four. He remained under surveillance, and CT four months postoperatively revealed local recurrence. Due to disease progression, he is currently receiving multidisciplinary treatment at another institution.

## Discussion

SCTs are rare urological lesions with an annual incidence of 0.3 cases per million people [4]. The majority of SCTs are benign; however, approximately 25% are malignant, primarily sarcomas [5]. Among malignant SCTs, liposarcoma is the most common histological type, followed by leiomyosarcoma and rhabdomyosarcoma [6].

Metastatic SCTs are even rarer, accounting for only 8.1% of all SCTs [2]. One explanation for their rarity is that the relatively low temperature of the SC may not be conducive to tumor cell implantation [7]. The most frequently reported primary sites of metastatic SCTs include the stomach, colon, pancreas, kidney, and prostate [1-3].

Several mechanisms have been proposed to explain this rare form of metastasis. The most commonly suggested pathway is retrograde lymphatic spread from the primary tumor site [8]. Peritoneal dissemination has also been proposed [9]. Furthermore, cases in which a histopathological examination revealed tumor thrombi suggest the possibility of hematogenous metastasis [10]. In the present case, transverse colon cancer was classified as pT4aN3 at the time of surgery, with lymphovascular invasion and venous invasion identified. Due to the presence of lymphovascular invasion, venous invasion and exposure to the peritoneal cavity during primary tumor resection, retrograde lymphatic spread, hematogenous spread, and peritoneal dissemination were all plausible mechanisms of metastasis.

The prognosis of patients with SC metastases is generally poor, with a reported median survival of 9.1 months after diagnosis [2]. However, previous studies suggested that patients with late-onset metastases, occurring more than six years after primary tumor resection, have a more favorable prognosis [1]. In the present case, SC metastasis occurred three years after resection of the primary tumor, followed by local recurrence four months after radical orchiectomy. Due to these findings, a poor prognosis is expected, highlighting the importance of multidisciplinary treatment strategies.

## Conclusions

Although metastatic SCTs are rare, their clinical significance cannot be underestimated due to their poor prognosis and limited survival after diagnosis. Therefore, in patients with a history of gastrointestinal cancer surgery, metastatic tumors need to be considered as a differential diagnosis of SC masses, even in the absence of elevated tumor markers. Early recognition, histopathological confirmation, and timely surgical intervention are crucial for optimizing outcomes. Greater awareness of this rare metastatic pattern may aid in earlier diagnosis and enhance clinical decision-making in similar cases.
